# The role of frequency and severity in measuring oral health-related quality of life: a cross-sectional study with a test–retest subsample in general and clinical populations

**DOI:** 10.1186/s12903-026-08172-2

**Published:** 2026-03-30

**Authors:** Judit Oszlánszky, Péter Hermann, Zsombor Zrubka

**Affiliations:** 1 Department of Prosthodontics, Faculty of Dentistry, Semmelweis University, Szentkirályi street 47, Budapest, Hungary; 2 Health Economics Research Center, University Research and Innovation Center, Obuda University, Budapest, 1034 Hungary

**Keywords:** GOHAI, Oral health, Quality of life, Psychometric, Recall consistency, PROM

## Abstract

**Background:**

We explored three key aspects of the Geriatric Oral Health Assessment Index (GOHAI): consistency of recalling the presence of problems across time intervals; test–retest reliability of frequency ratings; and the extent to which problem severity, frequency, and their combination explain general health-related quality of life (GHRQoL).

**Materials and methods:**

This cross-sectional study was conducted in Budapest between May 2023 and February 2024 in urban clinical and community settings, recruiting a mixed sample (*n* = 306) with diverse oral health status. GOHAI was re-administered after one week to 108 participants with stable oral health. When participants reported problems on any GOHAI item, they were asked whether these occurred in the past week, the past month, or whether they had persisted for more than three months. Recall inconsistencies were assessed across five time categories. Rating consistency was compared across three scenarios with different frequency response options. We also examined how problem severity and frequency contribute to OHRQoL: we predicted GHRQoL (EQ VAS) via frequency-based (GOHAI or OHIP-14) and severity-based (Oral Health Single-Question; OH-SQ) OHRQoL measures using multiple regression.

**Results:**

At least one problem was reported by 88.6% of respondents, and frequency ratings showed 15.4% inconsistency in the retest sample. Recall inconsistency ranged between 7.4% and 11.0% across time intervals and was lowest when no problems were present. Simple count ratings and the “presence within 3 months” criterion were more consistent than five-level frequency ratings. Compared to demographics alone (R² = 0.112), adding ADD-GOHAI improved EQ VAS prediction (R^2^ = 0.207, ΔR² = 0.095, *p* < 0.001), and adding OH-SQ further increased explained variance (R² = 0.267, ΔR² = 0.155; *p* < 0.001).

**Conclusion:**

Reducing response options and combining frequency- and severity ratings may enhance reliability and sensitivity. A one-week recall period appears optimal. These findings support the development of streamlined, patient-centered OHRQoL instruments.

**Supplementary Information:**

The online version contains supplementary material available at 10.1186/s12903-026-08172-2.

## Introduction

Henrietta L. Logan, a notable figure in quality-of-life research, highlighted a shift in healthcare expectations as early as 1997 [1]. Patients now seek more from dentists than just competence and reliability; they want involvement in their treatment, education about options, and personalized attention [2]. Over the past three decades, this demand has grown, especially with digital technology introducing new treatment alternatives and materials, along with varying costs. The Institute of Medicine defines patient-centred care as care that respects and responds to individual patient preferences, needs, and values [3]. A 2016 UK review noted that while patient-centred care is gaining attention in dentistry, it is less applied and studied than in general medicine. References to patient-centred care are more common in dental literature and policies, but a clear definition and practical implementation in dentistry are still lacking [4].

Studies often overlook the patient’s perspective when evaluating therapeutic solutions in dentistry [5]. With the rise of digital workflows, understanding how modern procedures affect dental patient-reported outcomes (PROs) has become increasingly important. Digital workflows, such as intraoral scanning and CAD/CAM technology, have been shown to reduce discomfort and enhance patient satisfaction, leading to improved dental PROs compared with conventional methods [6, 7]. A key dental PRO is Oral Health-Related Quality of Life (OHRQoL), encompassing orofacial function, pain, appearance, and psychosocial impact [8]. A 2021 study reviewed OHRQoL instruments validated for adults, identifying 42 original tools, with the 14-item Oral Health Impact Profile (OHIP-14) [9] and the General Oral Health Assessment Index (GOHAI) [10] as the most commonly used [11]. A 2024 review focused on OHRQoL tools for older adults, finding 14 instruments, of which 8 were generic and 6 were specific to treatments or conditions. The GOHAI and OHIP ranked second and third overall when using the Evaluating Measures of Patient-Reported Outcomes (EMPRO) tool [12]. This review also highlights that most OHRQoL instruments are not suitable for detecting changes in oral health in older adults. Similarly, Oszlánszky et al.‘s COnsensus-based Standards for the selection of health Measurement INstruments (COSMIN) review points out that the evidence on the GOHAI’s responsiveness is scarce, indicating the need for further research in this area [13].

The most widely used tools for measuring OHRQoL, including the GOHAI and OHIP-14, employ frequency-based Likert-type items ranging from 3 to 6 points. In such frequency-based scaling, response options reflect how often a problem occurs, whereas in severity rating, the focus is on how intense or bothersome the problem is. The frequency rating approach assumes that for a problem to impact quality of life, it must reach a certain level of severity. Frequency-based scales aim to capture aspects of OHRQoL by assuming that if a problem is present, it has already surpassed a threshold of severity, thereby making frequency assessment alone sufficiently informative. However, this method has its limitations. Many dental problems are inherently persistent, often lasting far beyond one, three, or even twelve months, and variations in their severity cannot be adequately captured using a frequency scale alone. Severity, in contrast, may capture the intensity of a problem’s impact on daily functioning and well-being, and thus may explain variance in OHRQoL even when frequency remains unchanged.

Other measurement tools, such as the different types of Oral Health Single Questions (OH-SQs), which are frequently used due to their simplicity, assess problems solely on a severity scale [14, 15]. This approach is similar to that used in general health-related quality of life (GHRQoL) questionnaires such as the EuroQol five-dimension five-level questionnaire (EQ-5D-5 L [16]).

This raises the logical question: would measuring both severity and frequency of problems provide the most comprehensive information on OHRQoL? Why don’t the most widely used questionnaires adopt this approach? The Oral Impacts on Daily Performances (OIDP), a self-administered questionnaire, attempts to combine the advantages of both measurement methods by evaluating severe oral impacts on daily activities, considering both frequency and severity of problems [17]. However, the additional information provided by the OIDP complicates score interpretation, increases the burden on respondents and administrators, and results in lower internal consistency and reproducibility [18].

When measuring problem frequency, a crucial question is determining the appropriate reference interval [19]. Unlike GHRQoL assessments that focus on the present moment, frequency-based responses require a longer reference period. However, intervals of three months or more may be too extended for effective personal follow-up, which is essential in patient-centred care. One of the main concerns is the reliability of patients’ memory and the consistency of their responses over different timeframes. For example, Sutinen et al. found no significant difference between one-month and twelve-month reference intervals for OHIP-14 responses [20]. The inaccuracies associated with longer time intervals lead to higher Smallest Detectable Change values, which limits the usefulness of these tools for individual monitoring. For instance, applying a three-month interval to the GOHAI yields a relatively high Smallest Detectable Change of 5 points on its 12–60 range [21].

From a measurement perspective, widely used OHRQoL instruments such as the GOHAI and the OHIP-14 are based on aggregated frequency ratings of multiple oral health impacts. This scoring approach is often interpreted as implicitly formative, as the total score is constructed from the presence and frequency of distinct problems. At the same time, these instruments are conceptually intended to assess oral health–related quality of life as a single underlying construct, which is more consistent with a reflective framework.

Consequently, identical GOHAI or OHIP-14 scores may result from different combinations of oral health problems, and their measurement properties may vary across populations. Previous psychometric evidence suggests that GOHAI exhibits both formative and reflective characteristics, while still supporting its interpretation as a one-dimensional instrument [13].

In contrast, severity-based measures such as the Oral Health Single Question provide a global, reflective assessment of oral health, focusing on how burdensome problems are for the individual rather than how often they occur. Given these conceptual differences, examining the contribution of frequency- and severity-based measures may deepen our understanding of the OHRQoL construct.

Given the limited evidence on how patients reconstruct symptom timelines, particularly for persistent symptoms, the consistency of temporal recall warrants closer examination. Motivated by these gaps and the open questions about the measurement properties of GOHAI, this study aimed to examine three aspects of GOHAI: (1) the recall consistency of the presence or absence of item-level problems across different time intervals; (2) the recall consistency of problem frequency ratings; and (3) the extent to which problem severity, frequency, and their combination explain variations in OHRQoL. We hypothesized that fewer response options and shorter recall periods would yield more consistent reporting of oral health problems, and that problem severity and frequency would explain better OHRQoL than each alone.

## Methods

Study design.

This was a cross-sectional study with a test–retest subsample. This study was part of the Hungarian GOHAI validation, involving a diverse sample by gender, age, and oral health status [21].

### Participants

Participants (*n* = 306) were recruited from a nursing home (*n* = 33), public screenings (*n* = 105), and three departments at Semmelweis University: Oral Diagnostics (*n* = 70), Temporomandibular Disorders Care Unit (*n* = 67), and Prosthodontics (*n* = 31). All patients underwent clinical oral examination by dental specialists. All questionnaires were administered in an interviewer-administered, paper-based format by the same trained oral medicine specialist (OJ), who also conducted all retest interviews; therefore, no further investigator training or calibration was required. For patients with stable oral health, the questionnaire was re-administered by the same doctor after one week, either in person (*n* = 62) or by phone (*n* = 46). Stable oral health was defined as no reported change in patients’ oral condition or OHRQoL, and verified through two control questions with opposite polarity.

Ethical approval was granted by Semmelweis University (permit no. 61/2023), and written informed consent was obtained from all participants prior to their participation.

The sample size was determined based on psychometric validation requirements, following the COSMIN-modified GRADE approach, which recommends a minimum of 100 participants per subgroup to ensure high-quality evidence for the evaluation of measurement properties. This justification also ensured that the retest subsample exceeded the commonly accepted minimum (*n* ≥ 100) for reliability analyses [22, 23]. 

**Fig. 1:**
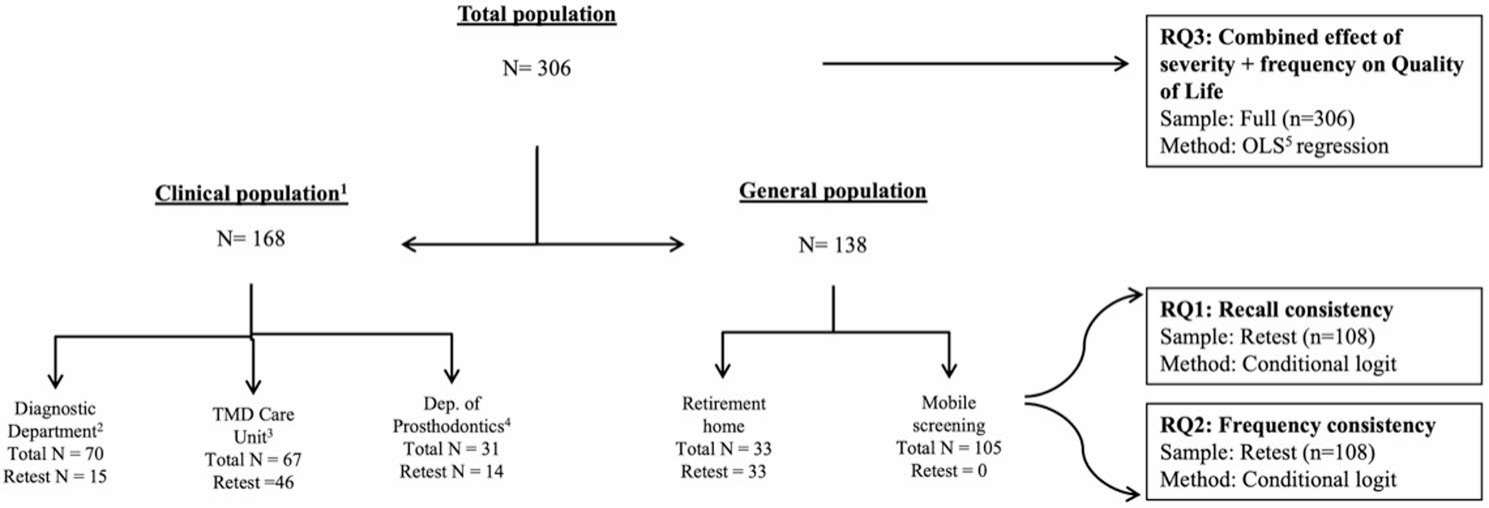
Recruitment flow and analytical structure of the study. ^1^Clinical population from Semmelweis University, Faculty of Dentistry’s outpatient services: ^2^ Department of Oral Diagnostics, ^3^ Temporomandibular Disorders Care Unit, ^4^ Department of Prosthodontics, ^5^ ordinary least squares regression

Figure 1 presents the study flow and analytical structure, showing recruitment sources, the composition of the full and retest subsamples, and the allocation of research questions (RQ1–RQ3) to each sample and analytical method. As this was a convenience sample without a formal screening log, only the participating population and the subset reached for retest are displayed.

### Questionnaire and scoring

The questionnaire included six sections: demographics; OH-SQ (measuring oral health status on a six-stage severity scale from 1 = excellent to 6 = very poor); an oral symptom checklist; the Hungarian GOHAI [21]; OHIP-14 [24]; and EQ-5D-5 L [16]. GOHAI items were rated on a 5-point Likert-type severity scale (1 = always, 5 = never) over the past three months. GOHAI was scored in two ways: the additive score (ADD-GOHAI) sums the five-level frequency ratings of each item (range: 12–60; higher scores indicate better OHRQoL), while the simple count score (SC-GOHAI) collapses responses into “never/seldom” vs. “sometimes/often/always,” counting the number of items in the latter category. Higher SC-GOHAI scores indicate worse OHRQoL.

Internal consistency of the GOHAI-HU had been previously established. Cronbach’s α values for all subgroups exceeded 0.70 for both ADD-GOHAI and SC-GOHAI, indicating adequate internal consistency; these values are reported here for completeness [21].

The EQ-5D-5 L includes a descriptive system covering problem severity in five levels across five health domains and a visual analogue scale (EQ VAS), which rates self-perceived health from 0 (worst) to 100 (best). EQ-5D-5 L index values were calculated using the Hungarian time-trade off value set. The EQ-5D-5 L index quantifies GHRQoL, based on preferences of the Hungarian general population, on a scale from − 0.848 (worst health state defined by the instrument) to 1 (perfect health), with 0 representing a health state equivalent to death [25]. A comprehensive overview of all measurement instruments, including their items, response formats, score ranges, and interpretation, is provided in Supplementary Table S8.

Time interval categories.

For each GOHAI item, we inquired if problems were present in the following time interval categories:

1. *Past 3 months*: the problem occurred at least once within the past three months (i.e., the “never” option was not selected).
2. *Last week*: the problem was present within the last week.
3. *Last month*: the problem occurred within the past month.
4. *Before 3 months*: the problem has been present for more than three months (i.e., it started earlier) but was also present within the past three months.
5. *Persistent*: the problem was present within the last week and has been ongoing for more than three months. This category was constructed by combining the “last week” and “before 3 months” responses. This category was defined a priori to capture long-standing symptoms that are both chronic in duration and currently active. While the “before 3 months” category reflects symptom onset and duration, the “persistent” category also accounts for current presence, thereby distinguishing ongoing, continuously experienced problems from those that may fluctuate or have resolved recently.

The retest survey included two questions assessing the perceived stability of oral health status and GOHAI-HU with the time interval questions.

### Statistical analysis

#### Sample characteristics

The sample characteristics relevant to the present study are presented in Table 1. For additional descriptive details about the broader validation sample, see the GOHAI-HU validation study [21]. Here, we briefly summarize main demographic variables and the mean (SD) values of ADD-GOHAI, SC-GOHAI, OHIP-14, OH-SQ, EQ-5D-5 L index, and EQ-VAS. Also, we provide the test-retest reliability of ADD-GOHAI scores measured via intraclass correlation coefficient (ICC) values.

#### Presence of problems across different time intervals

We calculated the percentage of reported problems per respondent across the 12 GOHAI items. For each item, where a problem was present (i.e., any response other than “never”), we assessed the percentage of problems occurring in four time intervals: past week, the past month, before 3 months, and persistent. A conditional logit model was used to compare the likelihood of reporting problems across time intervals. The conditional logit model takes into account that multiple responses (12 items × 4 time intervals) come from the same respondent [26]. The binary dependent variable was problem presence; the predictor was time interval, with the “past 3 months” as the reference category. Coefficients (odds ratios) were compared using Wald tests. The conditional logit models were run on the subsample without missing responses on GOHAI items. We also calculated item-level percentages of reported problems for each time interval and noted any missing responses.

#### Consistency of problem recall across time intervals

Problem recall was considered consistent if a respondent gave the same answer (problem present or absent) to an item at both administrations. We calculated the percentage of inconsistent responses per interval across the 12 GOHAI items. We then used a conditional logit model to compare the likelihood of inconsistent recall across the four time intervals, using “past 3 months” as the reference category. The conditional logit model accounts for the fact that inconsistency is observed multiple times (12 items x 4 time intervales) in the same respondent. For each time interval, we also assessed whether item-level inconsistency differed depending on whether a problem was reported as present or absent at first administration of GOHAI. The percentage of inconsistent responses by time interval was also calculated for each item.

### Consistency of problem frequency ratings

Frequency ratings were consistent if the same category was selected for an item at both administrations, and inconsistent otherwise. The percentage of inconsistent answers across the 12 items was compared for three GOHAI scoring scenarios: problem presence (yes/no), five-level frequency ratings of ADD-GOHAI, and SC ratings of SC-GOHAI. A conditional logit model was used with inconsistency as the binary dependent variable and scoring scenario as categorical predictor. The model accounted for repeated observations per respondent (12 items x 4 time intervals). Using a conditional logit model, we also compared inconsistency between items with and without problems at the first administration, accounting for repeated observations per respondent (12 items x 2 options).

### The contribution of problem severity ratings and frequency to OHRQoL

To examine the contribution of problem frequency and severity to OHRQoL, we used GHRQoL as an external criterion. Because OHRQoL is expected to contribute to the broader GHRQoL construct, we used ordinary least squares regression to test whether combining frequency-based (GOHAI or OHIP-14) and severity-based (OH-SQ) OHRQoL measures explain more variance of GHRQoL than each alone. Two model sets were built with EQ-VAS (models 1–6) and EQ-5D-5 L index (models 7–12) as outcomes. Baseline models (M1, M7) included age, sex, and education. Models added ADD-GOHAI (M2, M8), OH-SQ and its interaction with ADD-GOHAI (M3, M9), OHIP-14 (M4, M10), OHIP-14 plus OH-SQ and their interaction (M5, M11) and ADD-GOHAI and OHIP-14 with their interaction (M6, M12). The interaction terms capture whether the frequency or oral symptoms contribute differently to GHRQoL at different symptom severity levels (e.g., the more severe the oral health problems are, the stronger the effect of their frequency on GHRQoL may be). As the OH-SQ assesses oral health over the past week, we included only respondents who reported a problem on at least one GOHAI item during the past week. Model fit was compared via R² and likelihood-ratio tests. Additional explained variance was expressed as ΔR² versus the baseline model. Predicted GHRQoL values were visualized by OHRQoL levels. Analyses were conducted in Stata 17 [27].

## Results

### Sample characteristics

The total sample included 306 participants who responded to the survey at least once. The retest sample involved 108 respondents. Test–retest reliability of ADD-GOHAI did not differ by mode of retest administration. Participants retested in face-to-face interviews (*n* = 62; ICC = 0.93; 95% CI: 0.88–0.96) and by telephone (*n* = 46; ICC = 0.97; 95% CI: 0.94–0.98) showed comparable ICC values, with overlapping 95% confidence intervals. The sample characteristics are shown in Table 1. Compared to those respondents, for whom the questionnaire was administered only once, the retest sample had slightly better oral health status in terms of OH-SQ (mean difference 0.34, two-sided t-test *p* = 0.026). Other differences were not significant between the two groups. Mean EQ-VAS was 71.8 (SD 17.6), indicating moderately good average self-perceived health with substantial variability across participants.

**Table 1:** Sample characteristics

		Population					
		Total	Clinical	General	Retest total	Retest Clinical	Retest general
Sample	N	306	168	138	108	75	33
Females	%	71.2%	73.8%	68.1%	72.2%	73.3%	69.70%
Age (years)	mean (SD)	57.3 (20.4)	48.9 (18.4)	67.6 (17.9)	59.8 (23.4)	47.4 (16.8)	87.8 (4.4)
Education	Primary %	16.0%	14.3%	18.1%	5.6%	8.0%	0.0%
	Secondary %	43.1%	47.0%	38.4%	45.4%	49.3%	36.4%
	Tertiary %	40.9%	38.7%	43.5%	49.0%	42.7%	63.6%
Residence	City %	75.5%	58.3%	96.4%	70.4%	58.7%	97.0%
	Town %	16.7%	28.0%	2.9%	23.1%	33.3%	0.0%
	Rural %	7.8%	13.7%	0.7%	6.5%	8.0%	3.0%
ADD-GOHAI	mean (SD)	50.3 (8.6)	48.9 (8.6)	52.1 (8.3)	51.5 (8.2)	49.7 (8.5)	55.4 (5.9)
SC-GOHAI	mean (SD)	3.0 (2.7)	3.5 (2.7)	2.4 (2.5)	2.7 (2.7)	3.3 (2.8)	1.4 (1.9)
OHIP-14	mean (SD)	63.7 (8.5)	61.8 (9.5)	66.0 (6.3)	63.3 (9.0)	61.5 (10.0)	67.4 (3.8)
OH-SQ	mean (SD)	3.6 (1.3)	3.7 (1.2)	3.5 (1.3)	3.4 (1.3)	3.5 (1.3)	3.15 (1.4)
EQ-5D-5 L index	mean (SD)	0.87 (0.19)	0.91 (0.13)	0.84 (0.25)	0.87 (0.20)	0.92 (0.13)	0.76 (2.9)
EQ VAS	mean (SD)	71.8 (17.6)	73.8 (16.7)	69.4 (18.4)	72.6 (18.4)	74.9 (17.0)	67.6 (20.6)

### Presence of problems across different time intervals

**Fig. 2:**
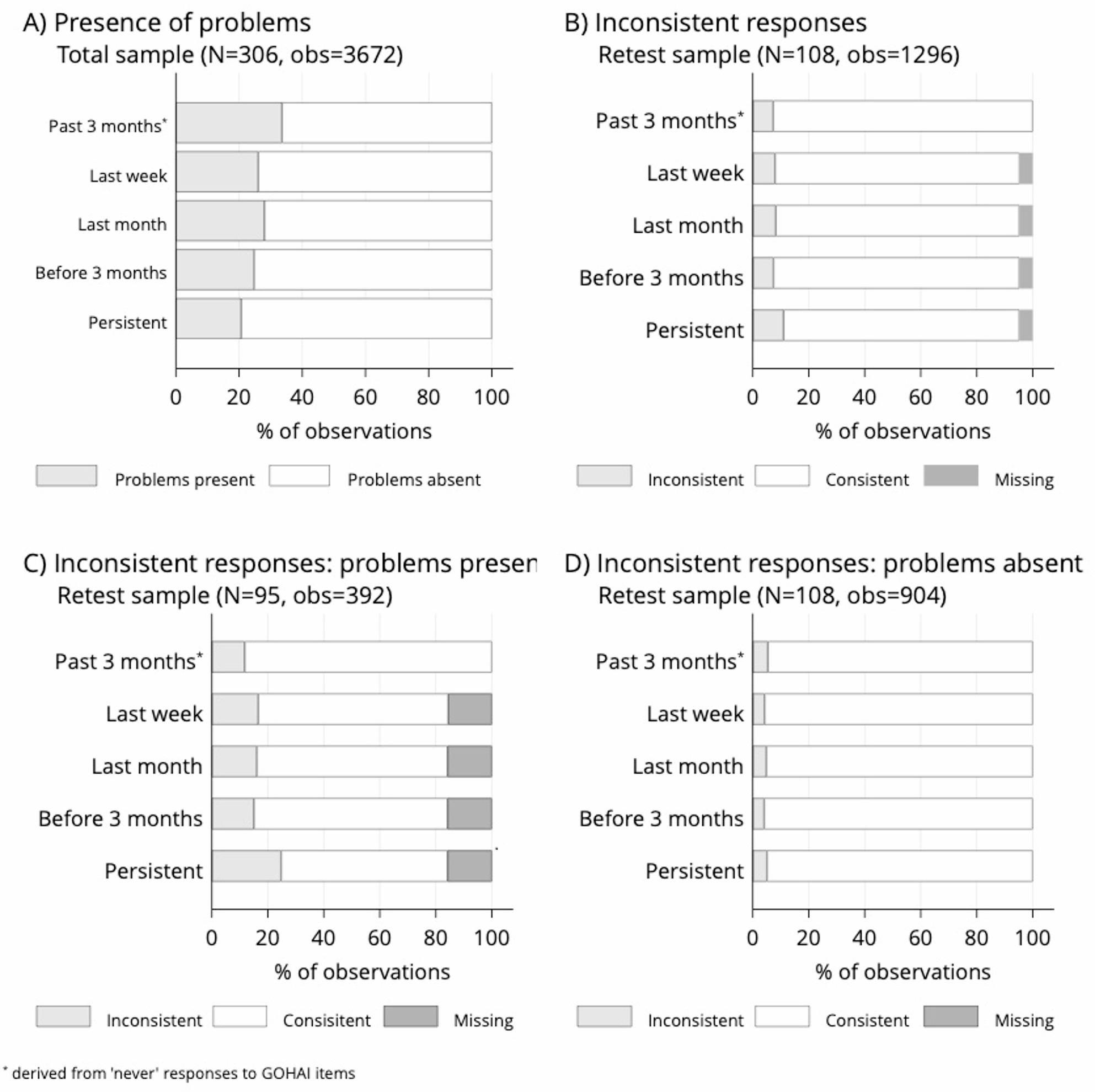
Recall of oral health problems across time intervals. Observations represent responses on 12 GOHAI items per respondent. **A** percentage of observations with problems reported in different time intervals; **B** percentage of observations with inconsistent responses about the presence of problems; **C** percentage of observations with inconsistent responses if problems were present at first survey administration; **D** percentage of observations with inconsistent responses if problems were absent at first survey administration

In total, 3672 observations (306 respondents × 12 GOHAI items) were analyzed per time interval. At least one present problem was reported by 88.6% of respondents (271/306). At first administration, problems were reported in 33.6% (1233/3672), 26.0% (955/3672), 27.9% (1026/3672), 24.7% (907/3672), and 20.7% (759/3672) of observations for the past 3 months, past week, past month, before 3 months, and persistent categories, respectively (Fig. 2A). Among problems reported for the past 3 months, 77.5% (955/1233), 83.2% (1026/1233), 73.6% (907/1233), and 61.6% (759/1233) also occurred in the past week, month, before 3 months, or persistently. All time intervals differed significantly from the “past 3 months” reference. Of problems present in the past month, 93.1% (955/1026) also occurred in the past week. The difference between the two intervals was significant. Coefficients and p values are provided in Table S1. Item-level details are shown in Figure S1A.

### Consistency of problem recall across time intervals

We analyzed 1296 observations from the retest sample (108 respondents × 12 GOHAI items); 95 participants reported at least one problem. Based on “never” responses, 7.3% (95/1296) of answers were inconsistent. The percentage of inconsistent and missing responses by time interval were respectively 7.9% (103/1296) and 4.7% (103/1296) in past week, 8.3% (107/1296) and 4.8% in past month, (62/1296), 7.4% (96/1296) and 4.8% (62/1296) before 3 months, and 11.0% (143/1296) and 4.8% (62/1296) if persistent (Fig. 2B). The percentage of inconsistent responses was similar across intervals, except for persistent problems, which showed more inconsistency (Table S2). Overall, responses were more consistent when the absence of problems was reported at first administration of GOHAI (Fig. 2C–D, Table S3). Item-level results are presented in Figure S1B, which indicates that long-standing symptoms are systematically recalled with lower temporal accuracy.

### Distribution of problem frequency ratings

**Fig. 3:**
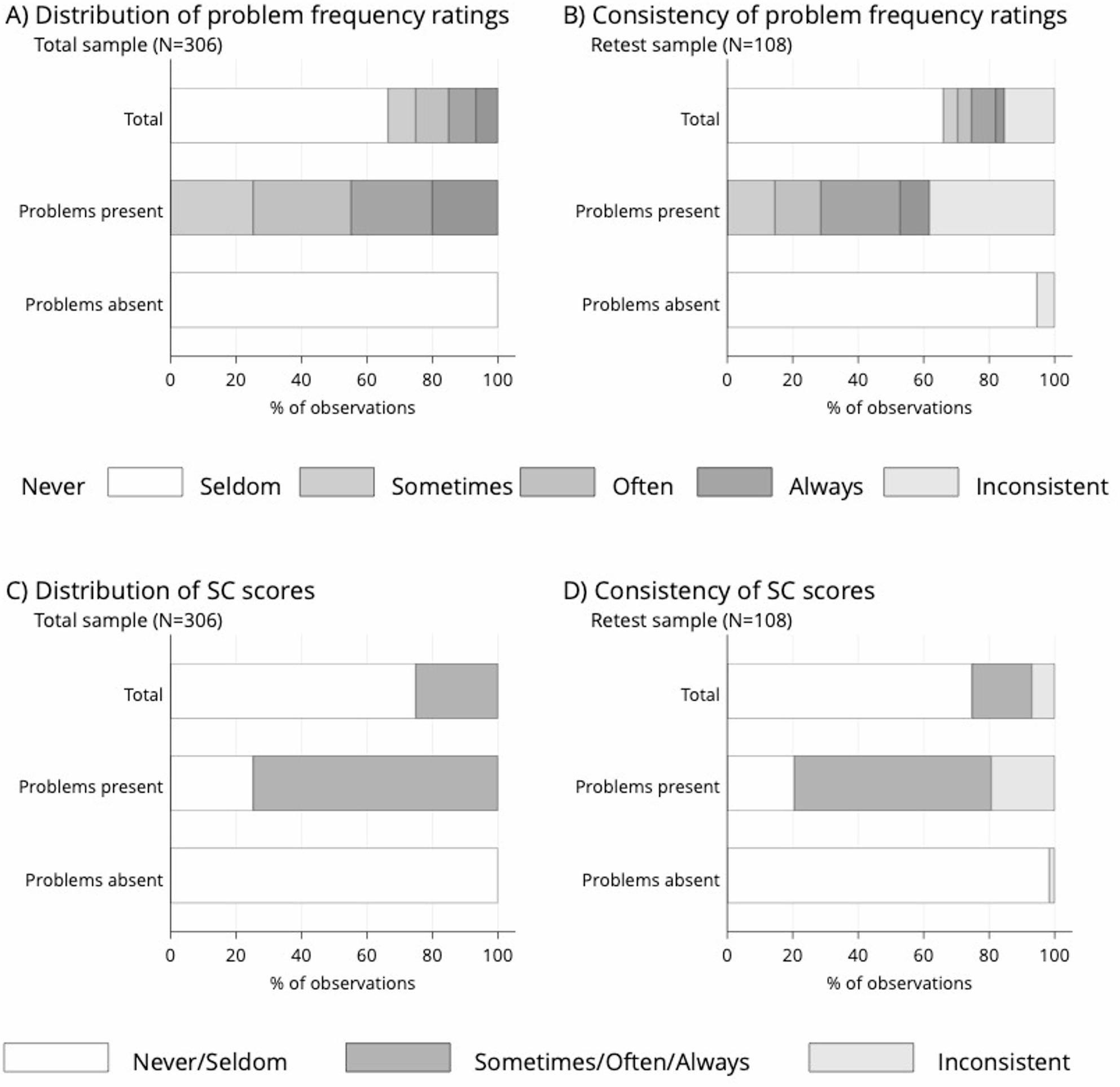
Frequency ratings and their test–retest consistency in the total sample, when problems were present and when problems were absent at first survey administration. Observations represent responses on 12 GOHAI items per respondent. **A** distribution of the five-level problem frequency ratings across all observations at first survey administration; **B** distribution of consistent and inconsistent five-level ratings of observations in the retest sample; **C** distribution of simple count (SC) scorings across observations at first survey administration; **D** distribution of consistent and inconsistent SC scorings across observations in the retest sample. **A **distribution of frequency ratings,** B** consistency of frequency ratings, **C** distribution of simple count ratings, and **D** consistency of simple count ratings

Of 3672 total observations, 33.6% (1233/3672) indicated no problems in the past 3 months. The percentages of frequency categories were: seldom 25.2% (311/1233), sometimes 29.9% (369/1233), often 24.8% (306 /1233), and always 20.0% (247/1233) (Fig. 3A). Using the SC rating, 25.1% (922/3672) of observations were rated as sometimes/often/always. Thus, 74.8% (922) of the 1233 problem reports were captured by the SC rating, while 25.2% (311) with “seldom” responses fell into the “never/seldom” category (Fig. 3C).

### Consistency of problem frequency ratings

In the retest sample (1296 observations), five-level frequency ratings were inconsistent in 15.4% (199/1296) of cases. Inconsistency was significantly more prevalent when problems were present (38.3%, 155/392) versus absent (5.4%, 49/904) at first administration (*p* = 0.001; Fig. 3B, Table S4). SC ratings showed lower inconsistency overall (7.0%, 91/1296), with 19.4% (76/392) inconsistency when problems were present and 1.7% (15/904) when absent (*p* < 0.001; Table S4). Table 2 summarizes the percentage of inconsistent responses by scoring scenario and initial problem presence. Odds ratios and p-values from conditional logit models are provided in Tables S4 and S5. SC and 3-month presence ratings were more consistent than five-level frequency ratings. Across all methods, the absence of problems was rated more consistently than their presence. If problems were present at first administration, the most consistent method was the 3-month problem presence rating. If problems were absent, the SC rating was most consistent. Five-level frequency ratings were the least consistent in all subgroups, indicating that simpler response formats yield more stable answers over time in clinical settings.

**Table 2:** Percentage of inconsistent responses by GOHAI scoring scenario and the presence or absence of problems during the first administration of GOHAI

	Total sample	Problems present at first administration of GOHAI	Problems absent at first administration of GOHAI
	% (*n*/*N*)	% (*n*/*N*)	% (*n*/*N*)
Presence of problems within 3 months	7.9% (103/1296)	11.7% (46 / 392)	5.4% (49 / 904)
Frequency ratings	15.4% (199/1296)	38.3% (155 / 392)	5.4% (49 / 904)
SC ratings	7.0% (91/1296)	19.4% (76/392)	1.7% (15 / 904)

Percentages of inconsistent responses are calculated for all available observations including individuals with missing responses on certain items. In Tables S4 and S5 of the appendix, odds ratios and *p* values are provided from conditional logit models using the subsample without missing responses on GOHAI items

### The contribution of severity ratings and frequency ratings to OHRQoL

Compared to demographics alone (M1), ADD-GOHAI (M2) significantly improved EQ-VAS prediction (R² from 0.112 to 0.207, ΔR²=0.095, *p* < 0.001), indicating that OHRQoL contributes substantially to variation in overall self-perceived health. Adding OH-SQ (M3) further increased the explained variance (R² = 0.267, ΔR²=0.155, *p* < 0.001), indicating that combining frequency- and severity-based OHRQoL measures better explains GHRQoL than frequency alone. This suggests that roughly one-quarter of the differences in EQ-VAS between individuals in our sample can be attributed to their oral health status. Results were similar when using OHIP-14 instead of GOHAI (M4, M5). However, combining OHIP-14 and GOHAI did not improve predictive power, suggesting that severity and frequency rating captures more information on OHRQoL than frequency rating alone (Table S6). Models using the EQ-5D-5 L index (M7–M12) showed similar trends, though with lower R² values (Table S7). 

**Fig. 4:**
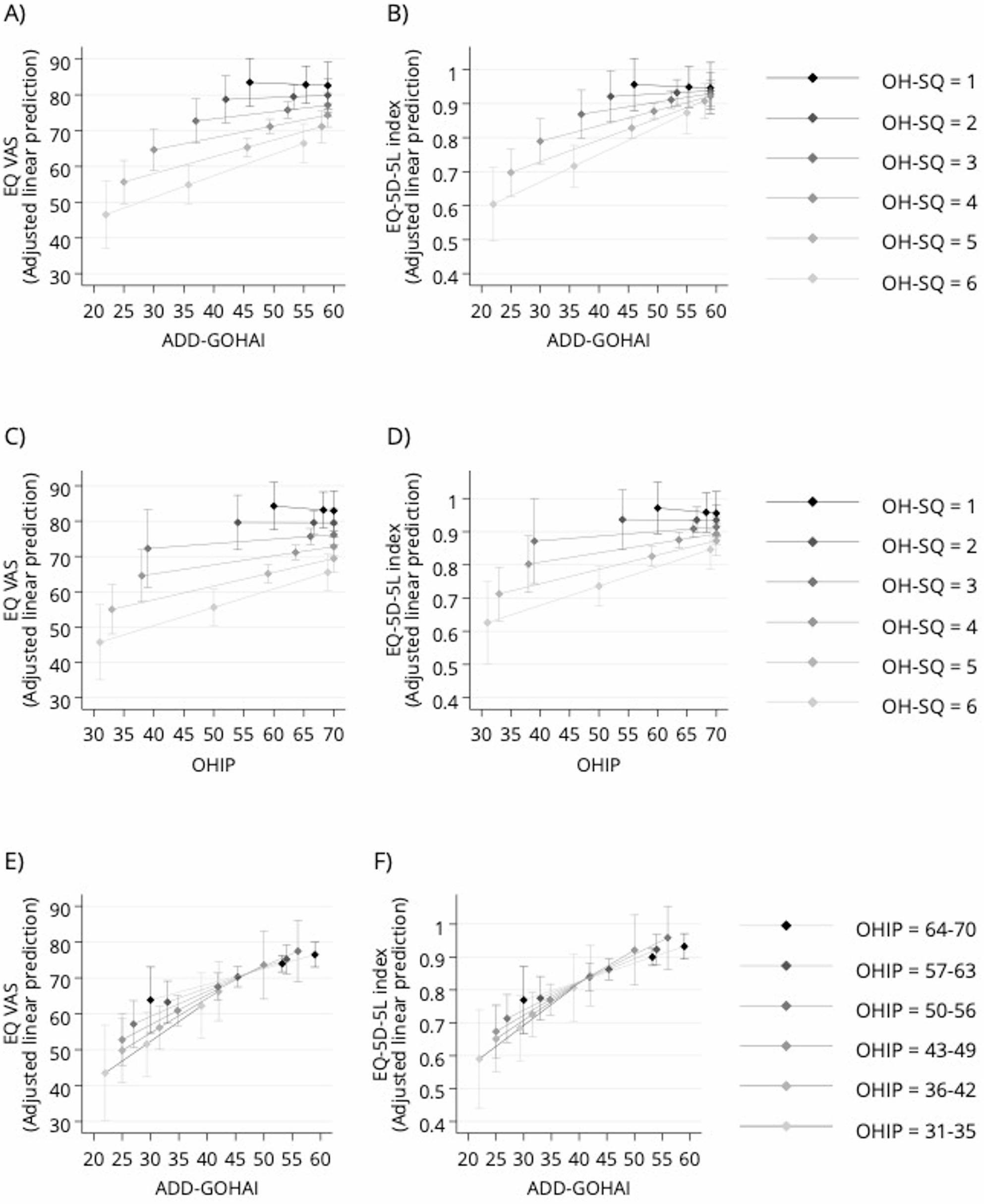
General Health-Related Quality of Life (GHRQoL) scores (EQ-VAS and EQ-5D-5L) explained frequency- and severity-based Oral Health-Related Quality of Life (OHRQoL) measures. **A**-**B** Predicted EQ-VAS and EQ-5D-5L index values by ADD-GOHAI scores at different OH-SQ severity levels; **C**-**D** Predicted EQ-VAS and EQ-5D-5L index values by OHIP scores at different OH-SQ severity levels; E-F) Predicted EQ-VAS and EQ-5D-5L index values by ADD-GOHAI scores at different OHIP score ranges

Figure 4 shows that the contribution of frequency-based OHRQoL scores (ADD-GOHAI, OHIP-14) to GHRQoL depends on problem severity. At low severity (OH-SQ = 1), frequency scores had no influence on EQ-VAS or EQ-5D-5L. At high severity (OH-SQ = 6), changes in problem frequency-based scores were associated with considerable GHRQoL changes (Fig. 4A–D). In contrast, the relationship between ADD-GOHAI and GHRQoL changed little across OHIP-14 levels (Fig. 4E–F).

## Discussion

Our principal findings indicate that symptom recall across time intervals was generally stable, with only 7.3% inconsistent responses. These results demonstrate that participants can reliably recall whether problems occurred, even when differentiating multiple timeframes. Novel and important contribution in our findings is the clear pattern that persistent, long-lasting symptoms were recalled less consistently. Responses were more consistent when no problems were reported, which supports the idea that the cognitive process of identifying the presence of a symptom is more robust than recalling its frequency.

The lower temporal consistency observed for long-standing symptoms may be related to cognitive mechanisms involved in symptom recall. For persistent conditions, respondents may rely on recall anchoring, whereby current symptom experience serves as a reference point and blurs distinctions between recent and more distant time periods. Telescoping effects may further contribute, leading individuals to misplace symptom onset or recurrence closer to the present.

In addition, symptom normalization may reduce temporal accuracy, as chronic problems become integrated into everyday functioning and are no longer perceived as discrete events tied to a specific timeframe. Together, these mechanisms may explain why persistent oral health problems are recalled with lower temporal precision, even when their presence is salient [28–30]. 

We also found that the simple count scoring method produced more consistent responses than the five-level additive scoring. While previous studies identified minimal differences between simple count scoring of GOHAI (SC-GOHAI) and the additive GOHAI score (ADD-GOHAI), using ICCs [31, 32], our findings clarify why: inconsistencies cluster primarily within the intermediate frequency categories. This directly supports the growing consensus that PROMs with fewer response options may reduce cognitive load and improve reliability [13, 33].

GOHAI scores explained significantly more variance in EQ-VAS than demographic variables alone, confirming that OHRQoL is an important component of GHRQoL. Adding the severity-based OH-SQ further increased explanatory power, suggesting that combining frequency- and severity-based measures captures the impact of oral health on overall quality of life better than either approach alone. The association between OHRQoL and GHRQoL became stronger as symptom severity increased, indicating an interaction between severity and frequency ratings. At mild problem levels, differences in OHRQoL (based on frequency ratings) had little effect on GHRQoL, whereas at severe problem levels, variation in OHRQoL was associated with notable differences in GHRQoL. Similar patterns were observed when either frequency-based OHRQoL instrument (GOHAI or OHIP-14) was combined with the severity-based OH-SQ. In contrast, combining two frequency ratings derived from frequency-based OHRQoL instruments did not improve the variance explained in GHRQoL.

This study has several strengths, including being the first to critically examine key aspects of the GOHAI, such as the consistency of symptom recall across time intervals, the stability of frequency ratings, and the combined role of symptom severity and frequency in explaining OHRQoL. Some limitations should also be noted. The predominantly urban, well-educated sample may limit generalizability to populations with lower literacy levels, rural communities, or those residing in low- and middle-income countries. Differences in cognitive load and cultural interpretations of symptom severity may influence recall accuracy and response consistency, so the applicability of our findings to these settings should be interpreted with caution. Although retest administration occurred via mixed modes (in-person or phone), supplementary analyses showed no evidence of mode effects on test–retest reliability. Regarding the retest interval, the retest occurred one week later, and participants’ oral health status was clinically stable, the time gap could have theoretically shifted some symptoms across recall categories. Still, the strong and highly significant consistency patterns suggest this had minimal impact on the findings. Although GOHAI was originally developed for older adults, its use has been extended to general adult populations for several decades and is supported by psychometric evidence across age groups. COSMIN-based systematic review findings and empirical validation studies, including the Hungarian GOHAI, confirm adequate reliability and construct validity in both younger and older adult populations [13, 21]. Accordingly, the findings of the present study are most directly applicable to adult populations, while the underlying measurement and recall processes examined are likely relevant across adulthood.

Our findings highlight several important directions for future research. First, recall period selection remains crucial for a PROM’s reliability and responsiveness. Very short timeframes may miss developing issues, while longer ones may dilute responsiveness. Peasgood (2023) demonstrated that patients typically report more severe symptoms and lower HRQoL when using a 7-day recall compared to a 1-day recall, suggesting that weekly recall captures symptom variability and infrequent experiences that single-day assessments may overlook. Patients in qualitative studies also expressed mixed preferences: while some preferred the accuracy of 1-day recall, others found a 7-day period more representative for fluctuating symptoms [30]. 

Waller (2016) compared 7-day and 1-month OHIP recall periods in stable prosthodontic patients and found that the two intervals demonstrated measurement invariance and equivalent validity in structural equation models, with similar associations to global oral health status. However, Waller emphasized that the conceptual advantages of a shorter (7-day) recall, such as better sensitivity to clinical change, make it preferable for clinical use [34]. 

Based on these insights and our results, a 1-week recall appears to represent a reasonable balance between accuracy, patient burden, and sensitivity to change. However, these results also suggest that recall-dependent frequency formats may introduce systematic measurement error, particularly for long-standing symptoms. We also hypothesize that fluctuations in severity may interfere with the accuracy of temporal recall of persistent problems, which should be tested in future research.

Further research should examine the psychometric effects of reducing response options. We observed better consistency with fewer categories, but it remains unclear whether collapsing five options into three yields the same results as designing a simpler scale from the outset.

Our results also underscore the value of including both frequency and severity dimensions in OHRQoL. Instruments relying solely on frequency apply a formative model, assuming equal item weight. Yet the same score may reflect vastly different health states, e.g., persistent mild discomfort versus acute pain. Prior work suggests OHRQoL instruments may have both formative and reflective features, supporting hybrid models [13]. For example, chronic mild orthodontic discomfort should not be equated with difficulty swallowing. Without proper weighting, scores may obscure clinical relevance. Nonetheless, increasing complexity with weighted scoring or mixed formats must be balanced against feasibility in clinical and research settings [12].

Evidence shows that pain, psychological distress, and functional limitation are distinct yet related components of OHRQoL, and symptom severity may similarly reflect multiple experiential constructs rather than a single intensity dimension [35]. Because these components can vary across individuals and cultural contexts, a unidimensional severity rating may mask meaningful differences and limit the generalizability of hybrid frequency–severity instruments. Future research should therefore examine whether differential weighting or multidimensional decomposition of severity offers a more valid basis for such hybrid PROM formats than assuming a uniform severity continuum.

Finally, to develop meaningful PROMs for patient-centred care, individual-level weighting of item importance should be explored. While societal value sets (e.g., in EQ-5D-5 L) provide average utilities for general use, capturing individual preferences remains a methodological challenge that warrants further study.

## Conclusion and practical implications

From a practical standpoint, our results suggest that OHRQoL assessment in routine care could be improved by: (1) adopting shorter recall periods (e.g., 1 week), (2) reducing response options, and (3) integrating both frequency and severity into a single hybrid score. For example, two patients may report a similar frequency of oral health problems yet differ markedly in perceived severity. While one patient may experience infrequent but highly bothersome symptoms requiring prompt intervention, another may report frequent but mild problems that warrant monitoring rather than immediate treatment. Incorporating severity alongside frequency can also reveal individual differences in symptom perception and patient priorities, thereby supporting more personalized clinical interpretation and treatment planning in routine dental care. These elements could be readily incorporated into chair-side digital tools or clinical software, enabling dentists to track patient outcomes more reliably and personalize treatment planning, in line with current best-practice for ePROM implementation in routine care [36, 37]. Future digital systems could build on these hybrid indices within computerized adaptive testing frameworks and, eventually, AI-assisted or emerging LLM-based PROM solutions that dynamically tailor item selection and interpretation to individual patients [38].

This research received no specific grant from any funding agency in the public, commercial, or not-for-profit sectors.

## Supplementary Information

Supplementary Material 1.

## Data Availability

The datasets used and analysed during the current study are available from the corresponding author on reasonable request.

## Abbreviations

ADD-GOHAI: Additive General Oral Health Assessment Index
EMPRO: Evaluating Measures of Patient–Reported Outcomes
EQ-5D-5L: EuroQol Five–Dimension, Five–Level Questionnaire
EQ VAS: EuroQol Visual Analogue Scale
GHRQoL: General Health–Related Quality of Life
GOHAI: General Oral Health Assessment Index
GOHAI-HU: Hungarian version of the General Oral Health Assessment Index
ICC: Intraclass Correlation Coefficient
OHRQoL: Oral Health–Related Quality of Life
OHIP-14: Oral Health Impact Profile, 14–item version
OH-SQ: Oral Health Single Question
OIDP: Oral Impacts on Daily Performances
OLS: Ordinary Least Squares (regression)
PRO: Patient–Reported Outcome
PROM: Patient–Reported Outcome Measure
SC: Simple Count
