# Supplementary material for: The role of frequency and severity in measuring oral health-related quality of life: a cross-sectional study with a test–retest subsample in general and clinical populations
Source: BMC Oral Health. 2026 Mar 30;26:808. doi: 10.1186/s12903-026-08172-2 (PMC13154905; doi:10.1186/s12903-026-08172-2)
Supplement: Supplementary file 1 — Supplementary Material 1. [file 12903_2026_8172_MOESM1_ESM.docx]

# **Appendix:**

**Psychometric Evaluation of the General Oral Health Assessment Index: The Role of Frequency and Severity in Measuring Oral Health-Related Quality of Life**

Judit Oszlánszky^1^, Péter Hermann^1^, Zsombor Zrubka^2^

^1^ Department of Prosthodontics, Faculty of Dentistry, Semmelweis University, Hungary

^2^ Health Economics Research Center, University Research and Innovation Center, Obuda University, Budapest, Hungary

Supplementary Table S1. Occurrence of problems in different time intervals, corresponding to the groups shown in Figure 1A of the manuscript

| Time interval | Coefficient  (Odds Ratio) ^†^ | (95%CI) | p |
| --- | --- | --- | --- |
| Base: vs. past 3 months |  |  |  |
| Past week | 0.62 | (0.55 - 0.69) | <0.001 |
| Past month | 0.70 | (0.63 - 0.79) | <0.001 |
| Before 3 months | 0.56 | (0.50 - 0.63) | <0.001 |
| Persistent | 0.42 | (0.37 - 0.47) | <0.001 |
| *N* | 16260* |  |  |

**271 respondents reporting at least one problem and no missing values were included in the analysis with 60 observations per respondent (12 GOHAI items × 5 time interval categories)*

*† Coefficients < 1 indicate lower probability of reporting problems vs. the base category*

Wald test of coefficients

Past week vs. past month: p=0.032

Past week vs. before 3 months: p=0.142

Past week vs. persistent: p<0.001

Past month vs. before 3 months: p=0.003

Past month vs. persistent: p<0.001

Before 3 months vs. persistent: p<0.001

Supplementary Table S2. Occurrence of inconsistent responses in different time intervals, corresponding to the groups shown in Figure 1B of the manuscript

| Time interval | Coefficient  (Odds Ratio)^†^ | (95%CI) | p |
| --- | --- | --- | --- |
| Base: vs. past 3 months |  |  |  |
| Past week | 1.10 | (0.81 - 1.49) | 0.535 |
| Past month | 1.15 | (0.85 - 1.56) | 0.356 |
| Before 3 months | 1.01 | (0.74 - 1.38) | 0.937 |
| Persistent | 1.65 | (1.24 - 2.21) | 0.001 |
| *N* | 4140* |  |  |

**Respondents reporting at least one problem and no missing values were included in the analysis with 60 observations per respondent (12 GOHAI items × 5 time interval categories)*

*† Coefficients > 1 indicate greater probability of inconsistent responses vs. the base category*

Wald test of coefficients

Past week vs. past month: p=0.762

Past week vs. before 3 months: p=0.588

Past week vs. persistent: p=0.005

Past month vs. before 3 months: p=0.398

Past month vs. persistent: p=0.011

Before 3 months vs. persistent: p=0.001

Supplementary Table S3. Comparison of inconsistent responses when problems were reported as absent or present during the first administration of GOHAI, corresponding to the groups shown in Figures 1C and 1D of the manuscript

|  | **Past 3 months** | | **Past week** | | **Past month** | | **Before 3 months** | | **Persistent** | |
| --- | --- | --- | --- | --- | --- | --- | --- | --- | --- | --- |
|  | Coeff. |  | Coeff. |  | Coeff. |  | Coeff. |  | Coeff. |  |
|  | (Odds Ratio)^†^  [95%CI] | p | (Odds Ratio)^†^  [95%CI] | p | (Odds Ratio)^†^  [95%CI] | p | (Odds Ratio)^†^  [95%CI] | p | (Odds Ratio)^†^  [95%CI] | p |
| Base: problem absent |  |  |  |  |  |  |  |  |  |  |
| Problem present | 2.30 | 0.001 | 6.66 | <0.001 | 4.65 | <0.001 | 5.26 | <0.001 | 7.57 | <0.001 |
|  | [1.41-3.74] |  | [3.89-11.4] |  | [2.81-7.70] |  | [3.06-9.04] |  | [4.73-12.1] |  |
| *N** | 648 |  | 627 |  | 653 |  | 675 |  | 771 |  |

** Respondents reporting at least one problem and no missing values were included in the analysis with 24 observations per respondent (12 GOHAI items × 2 subgroups)*

*† Coefficients > 1 indicate greater probability of inconsistent responses vs. the base category*

Supplementary Table S4. Comparison of inconsistent responses when problems were reported as absent or present during the first administration of GOHAI, corresponding to the inconsistent responses shown in Figures 2B and 2D of the manuscript

|  | | **Frequency ratings** | | **SC scores** | |
| --- | --- | --- | --- | --- | --- |
| Subgroup | Coefficient  (Odds Ratio)^†^  [95%CI] | p | Coefficient  (Odds Ratio)^†^  [95%CI] | p |  |
| Base: problem absent |  |  |  |  |  |
| Problem present | 2.30  [1.41-3.74] | 0.001 | 10.9  [5.95-20.1] | <0.001 |  |
| *N* | 648 |  | 600 |  |  |

** Respondents reporting at least one problem and no missing values were included in the analysis with 24 observations per respondent (12 GOHAI items × 2 subgroups)*

*† Coefficients > 1 indicate greater, <0 indicate smaller probability of inconsistent responses vs. the base category*

Supplementary Table S5. Inconsistent responses by three scoring scenarios of GOHAI

|  | **Total sample** | | **Problems present**  **at first administration of GOHAI** | | **Problems absent**  **at first administration of GOHAI** | |
| --- | --- | --- | --- | --- | --- | --- |
| Scoring scenario | Coefficient  (Odds Ratio)^†^  [95%CI] | p | Coefficient  (Odds Ratio)^†^  [95%CI] | p | Coefficient  (Odds Ratio)^†^  [95%CI] | p |
| Base: presence of problems within 3 months |  |  |  |  |  |  |
| Frequency ratings | 2.51 | <0.001 | 6.39 | <0.001 | 1.00 | >0.999 |
|  | [1.91-3.29] |  | [4.23-9.66] |  | [0.64-1.56] |  |
| SC ratings | 0.95 | 0.753 | 1.95 | 0.002 | 0.26 | <0.001 |
|  | [0.70-1.30] |  | [1.28-2.96] |  | [0.14-0.47] |  |
| *N* | 2916 |  | 1053 |  | 843 |  |

** Respondents reporting at least one problem and no missing values were included in the analysis with 36 observations per respondent (12 GOHAI items × 3 scoring scenarios)*

*† Coefficients > 1 indicate greater, <0 indicate smaller probability of inconsistent responses vs. the base category*

Wald test

Total sample: Frequency ratings vs. SC ratings: p < 0.001

Problems present: Frequency ratings vs. SC ratings: p < 0.001

Problems absent: Frequency ratings vs. SC ratings: p < 0.001Supplementary Table S6. Ordinary least squares (OLS) regression model explaining General Health-Related Quality of Life (GHRQoL) - measured by EQ VAS - by Oral Health-Related Quality of Life (OHRQoL) measures based on problem severity and frequency.

|  | **EQ-VAS** | | | | | |
| --- | --- | --- | --- | --- | --- | --- |
|  | M1 | M2 | M3 | M4 | M5 | M6 |
| Age | -0.257 | -0.247 | -0.226 | -0.265 | -0.233 | -0.249 |
|  | [-0.36,-0.16] | [-0.34,-0.15] | [-0.32,-0.13] | [-0.36,-0.17] | [-0.33,-0.14] | [-0.35,-0.15] |
|  | (<0.001) | (<0.001) | (<0.001) | (<0.001) | (<0.001) | (<0.001) |
|  |  |  |  |  |  |  |
| Education: secondary | 3.261 | -0.175 | -0.019 | 0.804 | 0.517 | -0.236 |
|  | [-2.75,9.27] | [-6.00,5.64] | [-5.64,5.60] | [-5.01,6.62] | [-5.07,6.11] | [-6.05,5.58] |
|  | (0.287) | (0.953) | (0.995) | (0.786) | (0.856) | (0.936) |
|  |  |  |  |  |  |  |
| Education: Tertiary | 8.029 | 3.659 | 2.947 | 4.473 | 3.200 | 3.470 |
|  | [2.00,14.06] | [-2.25,9.57] | [-2.78,8.67] | [-1.44,10.38] | [-2.50,8.91] | [-2.44,9.38] |
|  | (0.161) | (0.045) | (0.024) | (0.056) | (0.026) | (0.046) |
|  |  |  |  |  |  |  |
| Sex | 3.235 | 4.404 | 4.815 | 4.233 | 4.759 | 4.382 |
|  | [-1.30,7.77] | [0.09,8.71] | [0.65,8.98] | [-0.11,8.57] | [0.58,8.94] | [0.08,8.69] |
|  | (0.161) | (0.045) | (0.024) | (0.056) | (0.026) | (0.046) |
|  |  |  |  |  |  |  |
| ADD-GOHAI |  | 0.673 | -0.203 |  |  | 1.470 |
|  |  | [0.44,0.91] | [-1.06,0.65] |  |  | [-0.11,3.05] |
|  |  | (<0.001) | (0.642) |  |  | (0.069) |
|  |  |  |  |  |  |  |
| OHIP |  |  |  | 0.597 | -0.268 | 0.784 |
|  |  |  |  | [0.37,0.82] | [-25.00,-0.18] | [-0.20,1.77] |
|  |  |  |  | (<0.001) | (0.567) | (0.117) |
|  |  |  |  |  |  |  |
| OH-SQ |  |  | -10.680 |  | -12.593 |  |
|  |  |  | [-19.99,-1.37] |  | [-1.19,0.65] |  |
|  |  |  | (0.025) |  | (0.047) |  |
|  |  |  |  |  |  |  |
| OH-SQ*ADD-GOHAI |  |  | 0.134 |  |  |  |
|  |  |  | [-0.05,0.31] |  |  |  |
|  |  |  | (0.143) |  |  |  |
|  |  |  |  |  |  |  |
| OH-SQ*OHIP |  |  |  |  | 0.132 |  |
|  |  |  |  |  | [-0.06,0.32] |  |
|  |  |  |  |  | (0.174) |  |
|  |  |  |  |  |  |  |
| OHIP*ADD-GOHAI |  |  |  |  |  | -0.015 |
|  |  |  |  |  |  | [-0.04,0.01] |
|  |  |  |  |  |  | (0.208) |
|  |  |  |  |  |  |  |
| Intercept | 78.545 | 47.431 | 105.291 | 43.324 | 113.170 | 7.409 |
|  | [70.54,86.55] | [34.12,60.74] | [60.00,150.58] | [27.87,58.77] | [52.37,173.97] | [-50.90,65.72] |
|  | (<0.001) | (<0.001) | (<0.001) | (<0.001) | (<0.001) | (0.803) |
| N | 268 | 268 | 268 | 268 | 268 | 268 |
| R^2^ | 0.112 | 0.207 | 0.267 | 0.194 | 0.261 | 0.215 |
| LR test versus | - | M1 | M2 | M1 | M4 | M2 |
| LR test p value | - | (<0.001) | (<0.001) | (<0.001) | (<0.001) | (0.257) |

95% CI values in second row in square brackets

*p*-values are in thirds row in parentheses

Supplementary Table S7. Ordinary least squares (OLS) regression models explaining General Health-Related Quality of Life (GHRQoL) – measured by EQ-5D-5L index - by Oral Health-Related Quality of Life (OHRQoL) measures based on problem severity and frequency.

|  | **EQ-5D-5L index** | | | | | |
| --- | --- | --- | --- | --- | --- | --- |
|  | M7 | M8 | M9 | M10 | M11 | M12 |
| Age | -0.003 | -0.003 | -0.002 | -0.003 | -0.003 | -0.003 |
|  | [-0.004,-0.002] | [-0.004,-0.002] | [-0.004,-0.001] | [-0.004,-0.002] | [-0.004,-0.001] | [-0.004,-0.002] |
|  | (<0.001) | (<0.001) | (<0.001) | (<0.001) | (<0.001) | (<0.001) |
|  |  |  |  |  |  |  |
| Education: secondary | 0.043 | 0.007 | 0.007 | 0.020 | 0.017 | 0.005 |
|  | [-0.024,0.110] | [-0.059,0.072] | [-0.057,0.072] | [-0.046,0.085] | [-0.047,0.082] | [-0.060,0.070] |
|  | (0.209) | (0.844) | (0.825) | (0.555) | (0.597) | (0.878) |
|  |  |  |  |  |  |  |
| Education: Tertiary | 0.069 | 0.023 | 0.020 | 0.036 | 0.027 | 0.021 |
|  | [0.002,0.136] | [-0.043,0.089] | [-0.046,0.085] | [-0.031,0.102] | [-0.039,0.093] | [-0.045,0.088] |
|  | (0.044) | (0.496) | (0.553) | (0.294) | (0.427) | (0.523) |
|  |  |  |  |  |  |  |
| Sex | -0.006 | 0.006 | 0.008 | 0.003 | 0.007 | 0.005 |
|  | [-0.057,0.044] | [-0.042,0.054] | [-0.039,0.056] | [-0.046,0.052] | [-0.042,0.055] | [-0.043,0.053] |
|  | (0.803) | (0.808) | (0.731) | (0.905) | (0.785) | (0.829) |
|  |  |  |  |  |  |  |
| ADD-GOHAI |  | 0.007 | -0.003 |  |  | 0.018 |
|  |  | [0.004,0.010] | [-0.012,0.007] |  |  | [0.001,0.036] |
|  |  | (0.000) | (0.603) |  |  | (0.042) |
|  |  |  |  |  |  |  |
| OHIP |  |  |  | 0.006 | -0.003 | 0.007 |
|  |  |  |  | [0.003,0.008] | [-0.267,0.019] | [-0.004,0.018] |
|  |  |  |  | (<0.001) | (0.574) | (0.232) |
|  |  |  |  |  |  |  |
| OH-SQ |  |  | -0.114 |  | -0.124 |  |
|  |  |  | [-0.220,-0.007] |  | [-0.014,0.008] |  |
|  |  |  | (0.037) |  | (0.090) |  |
|  |  |  |  |  |  |  |
| OH-SQ*ADD-GOHAI |  |  | 0.002 |  |  |  |
|  |  |  | [-0.000,0.004] |  |  |  |
|  |  |  | (0.089) |  |  |  |
|  |  |  |  |  |  |  |
| OH-SQ*OHIP |  |  |  |  | 0.001 |  |
|  |  |  |  |  | [-0.001,0.004] |  |
|  |  |  |  |  | (0.186) |  |
|  |  |  |  |  |  |  |
| OHIP*ADD-GOHAI |  |  |  |  |  | -0.000 |
|  |  |  |  |  |  | [-0.000,0.000] |
|  |  |  |  |  |  | (0.192) |
|  |  |  |  |  |  |  |
| Intercept | 0.984 | 0.656 | 1.229 | 0.653 | 1.310 | 0.237 |
|  | [0.895,1.073] | [0.507,0.805] | [0.710,1.747] | [0.479,0.828] | [0.607,2.013] | [-0.416,0.890] |
|  | (<0.001) | (<0.001) | (<0.001) | (<0.001) | (<0.001) | (0.475) |
| N | 268 | 268 | 268 | 268 | 268 | 268 |
| R^2^ | 0.094 | 0.182 | 0.206 | 0.154 | 0.184 | 0.187 |
| LR test versus | - | M6 | M7 | M6 | M10 | M7 |
| LR test p value | - | (<0.001) | (0.017) | (<0.001) | (0.008) | (0.415) |

*p*-values are in second row in parentheses

Supplementary Figure S1. A) the presence of problems, and B) the frequency of inconsistent ratings in various time intervals by each GOHAI item


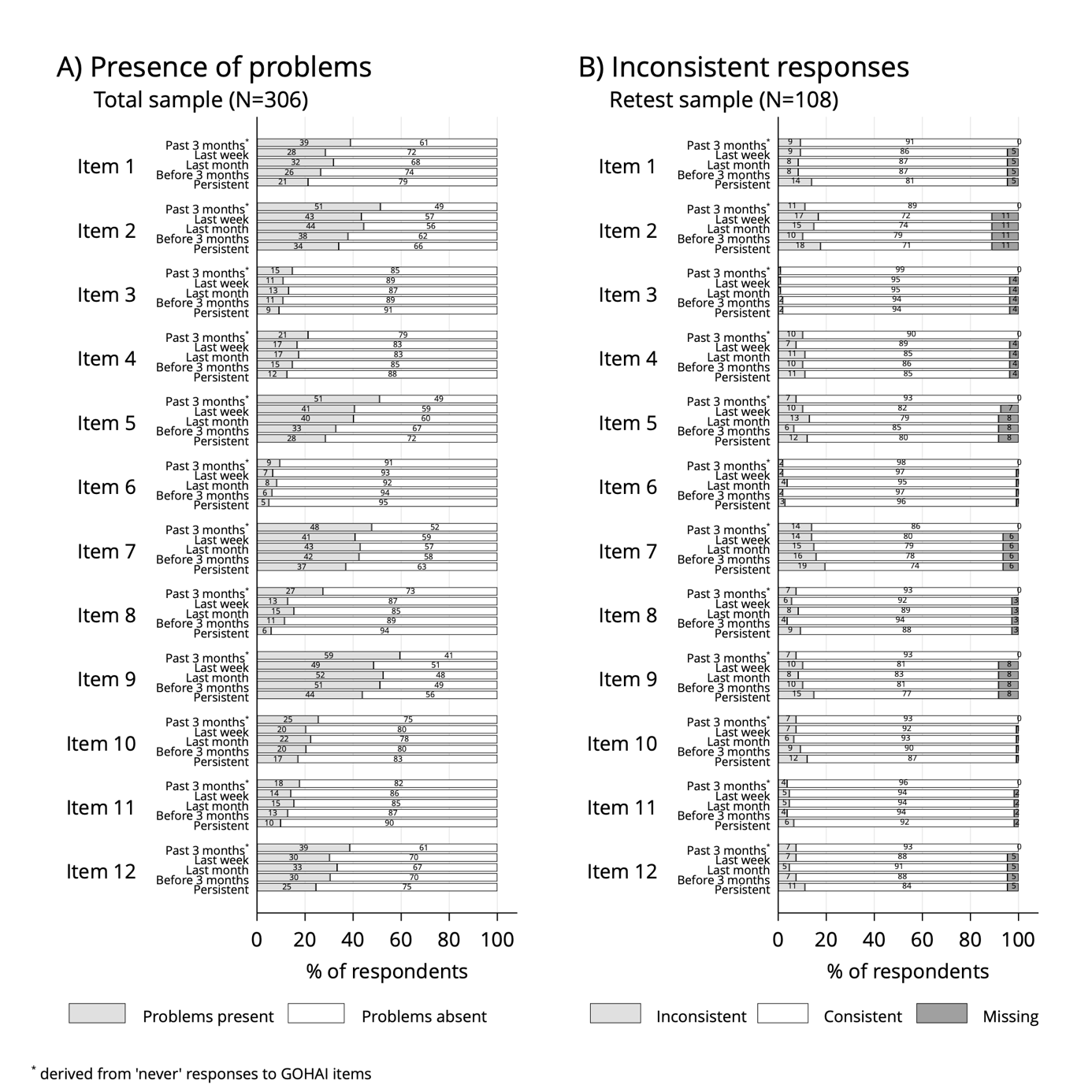


Supplementary Table S8. Overview of Measurement Instruments Used in the Study

| **Instrument** | **Type / Purpose** | **Number of items** | **Response options** | **Score range** | **Interpretation** |
| --- | --- | --- | --- | --- | --- |
| GOHAI | OHRQoL (frequency-based) | 12 | 5-point Likert (1=always, 5=never) | 12–60 | Higher = better OHRQoL |
| ADD-GOHAI | Summed frequency score | 12 | Same as GOHAI | 12–60 | Higher = better OHRQoL |
| SC-GOHAI | Binary simple count | 12 | Never/seldom vs sometimes/often/always | 0–12 | Higher = worse OHRQoL |
| OHIP-14 | OHRQoL (impact-based) | 14 | 5-point Likert (0–4) | 0–56 | Higher = worse OHRQoL |
| OH-SQ | Global oral health severity | 1 | 1–6 severity scale | 1–6 | Higher = worse oral health |
| EQ-5D-5L descriptive system | General HRQoL | 5 | 5 severity levels | — | Higher levels = worse problems |
| EQ-5D-5L index value | Health utility index | Composite | — | -0.86 to 1.00 | Higher = better HRQoL |
| EQ-VAS | Self-rated health | 1 | 0–100 scale | 0–100 | Higher = better health |
